# Crystal structures of thiamine monophosphate kinase from *Acinetobacter baumannii* in complex with substrates and products

**DOI:** 10.1038/s41598-019-40558-x

**Published:** 2019-03-13

**Authors:** Amy H. Sullivan, David M. Dranow, Peter S. Horanyi, Donald D. Lorimer, Thomas E. Edwards, Jan Abendroth

**Affiliations:** 1 UCB/Beryllium Discovery, 98110 7869 NE Day Road West, Bainbridge Island, WA USA; 2 Seattle Structural Genomics Center for Infectious Disease, Seattle, WA USA

## Abstract

Thiamine monophosphate kinase (ThiL) catalyzes the last step of thiamine pyrophosphate (TPP) synthesis, the ATP-dependent phosphorylation of thiamine monophosphate (TMP) to thiamine pyrophosphate. We solved the structure of ThiL from the human pathogen *A. baumanii* in complex with a pair of substrates TMP and a non-hydrolyzable adenosine triphosphate analog, and in complex with a pair of products TPP and adenosine diphosphate. High resolution of the data and anomalous diffraction allows for a detailed description of the binding mode of substrates and products, and their metal environment. The structures further support a previously proposed in-line attack reaction mechanism and show a distinct variability of metal content of the active site.

## Introduction

Thiamine pyrophosphate (TPP) is the biologically active form of vitamin B1 (thiamine) and is essential for all living organisms. Humans must obtain thiamine from their diet, whereas it can be synthesized *de novo* in bacteria, fungi and plants. TPP is a cofactor for multiple enzymes such as pyruvate dehydrogenase, transketolase, and alpha-ketoglutarate dehydrogenase. These enzymes are essential to carbohydrate metabolism and ATP synthesis^1,2^.

During biosynthesis of TPP, the thiazole and pyrimidine moieties of TPP are synthesized in two separate pathways. Thiamine phosphate synthase (ThiE) condenses these two building blocks to form thiamine monophosphate (TMP). Thiamine monophosphate kinase (ThiL) then catalyzes the ATP-dependent phosphorylation of TMP to form TPP^1^, Fig. S1. The biosynthesis of TPP is regulated by the TPP riboswitch which is found in both eukaryotes and prokaryotes^3^. Humans lack ThiL, potentially making it an attractive antimicrobial therapeutic target for pathogens.

The mission of the NIAID-funded Seattle Structural Genomics Center for Infectious Disease (SSGCID) is to provide the scientific community with protein structures of pathogens from NIAID category A-C agents, emerging and re-emerging infections disease organisms. These structures may be useful for drug or vaccine development, or may contribute to better understanding the virulence, pathogenesis, or markers of infection. The Gram-negative bacterium *Acinetobacter baumannii* has been classified as one of the ESKAPE pathogens (*Enterococcus faecium*, *Staphylococcus aureus*, *Klebsiella pneumonia*, *Acinetobacter baumanii*, *Pseudomonas aeruginosa*, and *Enterobacter* species), all of which have a high rate of antibiotic resistance, are opportunistic, and are responsible for a large number of hospital borne infections^4,5^.

The genomic sequence of a drug resistant clinical isolate of *Acinetobacter baumannii* AB5075-UW has recently been published^6^. The same study also identified genes essential for growth on nutrient agar using high-density transposon mutagenesis, and found thiamine monophosphate kinase (ThiL) to be essential. Structures of ThiL from *Aquifex aeolicus* (*Aa*ThiL, PDB ID 3C9R)^7^ and *Methylobacillus flagellatus* (*Mf*ThiL, PDB ID 3MCQ, no primary citation) have been described in the literature; however, no ThiL structures from pathogens are known.

In this study, we present high-resolution crystal structures of ThiL from the pathogen *Acinetobacter baumannii* (*Ab*ThiL) bound to substrate Thiamine monophosphate (TMP) and substrate analog adenylyl-imidodiphosphate (AMPPNP), and bound to its products TPP and adenosine diphosphate (ADP). The structures highlight both global structural differences and differences regarding ligand binding between *Ab*ThiL and comparable structures from Aquifex aeolicus (*Aa*ThiL)^7^. Anomalous diffraction could also confirm a plasticity of *Ab*ThiL with regards to metal identity in the active site. The structures further support proposals made on the reaction mechanism for *Aa*ThiL^7^.

## Material and Methods

### Cloning, expression, and purification

The gene for *Ab*ThiL (strain AB5075-UW, GenBank AKA29887.1, UniProt A0A0D5YC82) was amplified from genomic DNA and cloned into the expression vector pBG1861 using ligand-independent cloning^8^. The expression vector provides a non-cleavable N-terminal His_6_-tag (SSGCID target ID AcbaC.17905.a, SSGCID construct ID AcbaC.17905.a.B1, SSGCID batch AcbaC.17905.a.B1.PW37686). *Ab*ThiL was expressed in *E. coli* Rosetta BL21(DE3)R3 following standard SSGCID protocols as described previously^9^. Purification was done using Ni-NTA affinity and size exclusion chromatography following standard SSGCID protocols^10^. The purified protein was concentrated to 50 mg/ml in its final buffer (25 mM HEPES pH 7.0, 500 mM NaCl, 5% glycerol, 2 mM DTT, 0.025% NaN_3_), flash frozen in liquid nitrogen and stored at −80 °C.

### Crystallization

All crystallization experiments were done in 96-well XJR trays (Rigaku Reagents) with 0.4 µL protein diluted with final buffer to 25–30 mg/ml and 0.4 µL reservoir solution as sitting drops, equilibrated against 80 µL reservoir. Crystallization conditions were searched for by using the sparse matrix screens JCSG+ (Rigaku Reagents), MCSG1 (Microlytic/Anatrace), and Morpheus (Molecular Dimensions). Crystallization trays were incubated at 287 K. Crystals appeared in several conditions.

Initially, *Ab*ThiL was co-crystallized with 5 mM AMPPNP/MgCl_2_. Crystals from condition JCSG+ G10 (20% w/v PEG 2000MME, 150 mM NaBr) were cryoprotected in two steps with reservoir with 15% v/v ethylene glycol (EG), 5 mM AMPPNP/MgCl_2_ added, and vitrified by plunging them in liquid nitrogen.

Crystals from the same set up were used for experimental phasing: Crystal from JCSG+ B2 (20% w/v PEG 3350, 200 mM NaSCN) were soaked in two steps with reservoir with 10% v/v EG/250 mM NaI and 20% v/v EG/500 mM NaI for 30 sec each before vitrification.

Crystals grown in the presence of 5 mM AMPPNP/TMP/MgCl_2_ had many growth defects. Hence, co-crystals of *Ab*ThiL with 5 mM AMPPNP/MgCl_2_ from an optimization screen based on MCSG1 G4 (19% w/v PEG 3350, 200 mM K/Na-tartrate, 100 mM HEPES/NaOH pH 7.5) were soaked overnight with 5 mM AMPPNP/TMP/MgCl_2_. Crystals were harvested in two steps with reservoir with 20% v/v EG, 5 mM AMPPNP/TMP/MgCl_2_ added as the cryo protectant.

Co-crystallization of *Ab*ThiL with 5 mM ADP/TPP/MgCl_2_ yielded two crystal forms: Monoclinic crystals were obtained from Morpheus E5 (30 mM of each diethylene glycol, triethylene glycol, tetraethylene glycol, pentaethylene glycol; 100 mM Imidazole/MES monohydrate (acid); 40% v/v PEG 500MME, 20% PEG w/v 20,000), and harvested directly without additional cryo-protection. Orthorhombic crystals were obtained from MCSG1 B1 (20% w/v PEG 4000, 600 mM NaCl, 100 mM MES/NaOH pH 6.5) and were harvested with reservoir with 20% v/v ethylene glycol, 5 mM ADP/TPP/MgCl2 added as cryo protectant.

### Data collection and structure solution

Most of the data sets were collected in-house on a Rigaku FR-E^+^ 007 SuperBright rotating anode equipped with Rigaku VariMax optics and a Saturn 944+ detector, using CuKα X-rays. The AMPPNP/TMP data set was collected at the APS LS-CAT beamline 21-ID-F equipped with a C(111) monochromator, and a Rayonix MX-225 detector.

All data sets were reduced with the XDS package^11^ (Table 1). Only for the iodide data set, Friedel pairs were kept separate. For the AMPPNP, the AMPPNP/TMP, and the ADP/TPP data sets, Friedel mates were merged for the data set used for refinement; for the calculation of anomalous maps that were used to validate metals, Friedel mates were kept separate.

**Table 1:** X-ray data and refinement statistics for *Ab*ThiL.

Crystal parameters	Iodide	AMPPNP, orthorhombic	AMPPNP-TMP, orthorhombic	ADP-TPP, orthorhombic	ADP-TPP monoclinic
Space group	*P*2_1_2_1_2	*P*2_1_2_1_2	*P*2_1_2_1_2	*P*2_1_2_1_2	*P*2_1_
Cell dimensionsa = b = c (Å),α = β = χ (°)	86.95, 93.60, 72.49, 90, 90, 90	87.14, 93.76, 72.49, 90, 90, 90	86.96, 93.29, 73.48 90, 90, 90	87.15, 93.92, 73.70 90, 90, 90	50.75, 117.12, 55.89 90, 108,57, 90
**Data set**					
X-ray Source	Rigaku FRE+	Rigaku FRE+	APS 21-ID-F	Rigaku FRE+	Rigaku FRE+
Wavelength (Å)	1.5418	1.5418	0.97872	1.5418	1.5418
Resolution (Å)	50-2.0 (2.05-2.00)	50-1.75 (1.80-1.75)	50-1.70 (1.74-1.70)	50-1.90 (1.95-1.90)	50-1.55 (1.59-1.55)
Rmerge	0.062 (0.266)	0.051 (0.577)	0.053 (0.587)	0.047 (0.524)	0.062 (0.376)
I/sigma (I)	44.2 (10.0)	33.7 (3.9)	22.6 (3.4)	25.5 (2.7)	24.5 (3.6)
CC (1/2)	100.0 (98.2)	99	99.9 (87.1)	99.9 (78.4)	99.9 (88.2)
Completeness	99.4% (100%)	99.8% (99.9%)	99.5% (99.2%)	99.6% (97.5)	99.5% (94.8%)
# reflections overall	1,616,237 (64,146))	860,511 (36,501)	407,322 (29,982)	254,507 (10,998)	991,836 (6,382)
# reflections, unique	76,802 (5,726)	60,460 (4,402)	66,088 (4,824)	48,359 (3,421)	89,068 (6,266)
Multiplicity	21.0 (11.2)	13.3 (8.3)	6.1 (6.2)	5.3 (3.2)	11.1 (4.2)
SigAno	1.72 (0.90)	0.89 (0.74)	n/a	n/a	1.02 (0.75)
**Phasing statistics**					
FOM (PHASER)	0.39	—	—	—	—
FOM (PARROT)	0.74	—	—	—	—
**Refinement statistics**					
Rwork	—	0.1589	0.1495	0.1641	0.1419
Rfree	—	0.1936	0.1769	0.2124	0.1674
RMSD bond lengths (Å)	—	0.006	0.006	0.007	0.006
RMSD bond angles (**°**)	—	1.02	0.837	0.874	1.09
Ramachandran:	—				
preferred (%)	—	98.0%	97.4%	97.2%	97.8%
allowed (%)	—	2.0%	2.6%	2.6%	2.2%
disallowed	—	0.0%	0.0%	0.2%	0.0%
Molprobity clash score	—	1.78	2.21	2.787	2.25
Molprobity score	—	0.94	1.11	1.33	1.05
PDB code	—	5CC8	5DD7	6MFM	5CM7

For the iodide soaked crystal, 89 anomalous sites were found using HySS^12^ using data up to 2.1 Å resolution. The anomalous sites were further refined, and initial phases were calculated with Phaser_EP^13^ within the CCP4 package^14^. The CCP4 program PARROT^15^ was used for phase improvement; NCS averaging was not used due to low NCS correlations. An initial model was built with ARPwARP^16^.

### Structure refinement and validation

**Figure 1:**
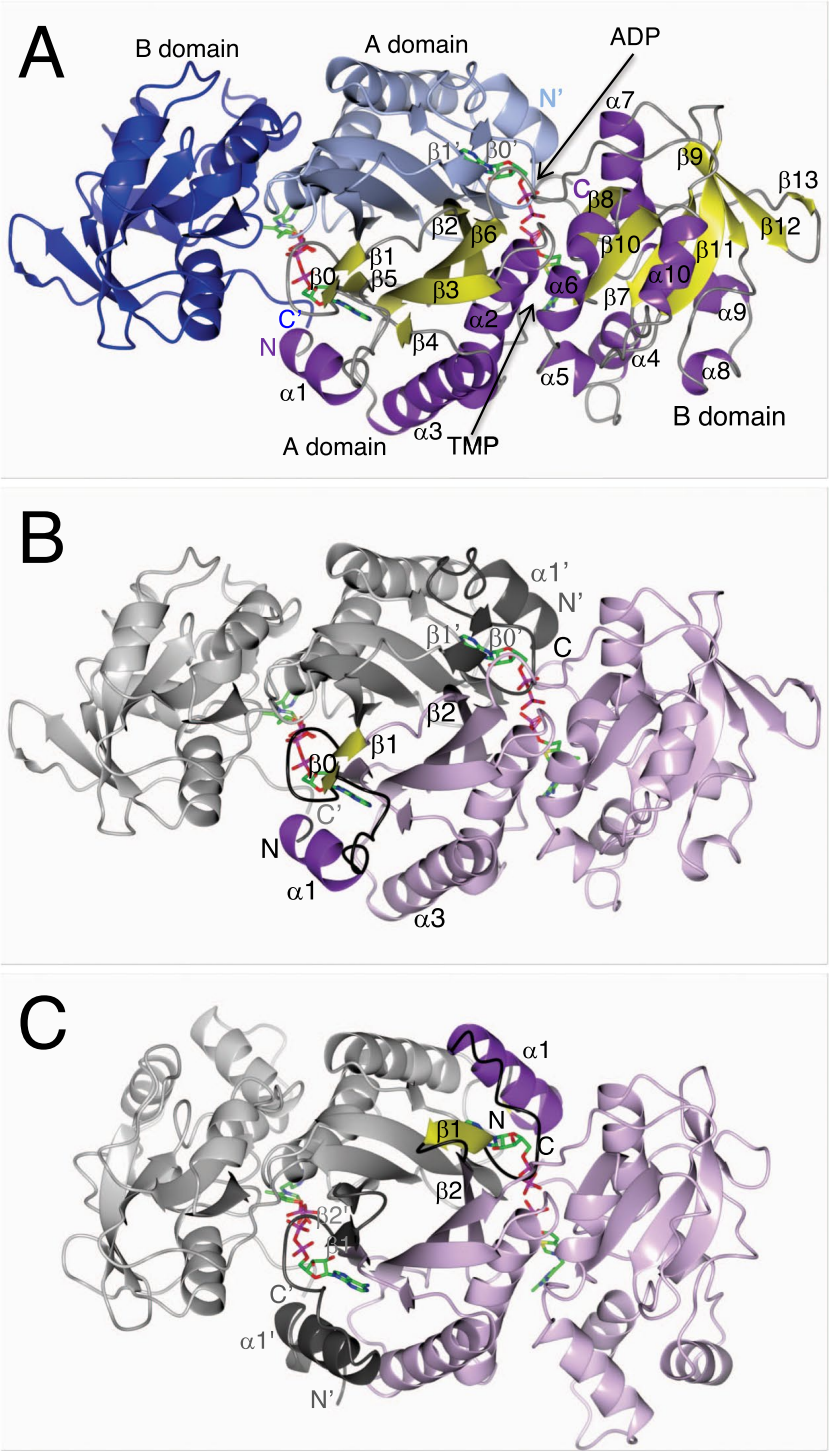
Dimer of ThiL. (**A**) shows the dimer of *Ab*ThiL bound to ADP/TMP looking down a dyad. The model of chain A on the right side is colored by secondary structure elements, helices in purple, strands in yellow. The model of chain B on the left side is colored by domains, the A domain in light blue, the B domain in blue. Strands β0′ and β1′ are part of the β-sheet that is mostly located in domain A. (**B**,**C**) highlight the domain swap of the N-terminus. In (**B**), the dimer of *Ab*ThiL is shown in the same orientation as in. (**A**) Chain A is colored in light purple with secondary structure elements α1, β0 and β1 highlighted in purple and yellow, respectively, and loops between them in black. The corresponding region in chain B is marked in dark grey. In (**C**), the model of *Aa*ThiL is shown in the same orientation and the same color scheme. Helix α1 and strand β1 of *Aa*ThiL are located in the space that helix α1′ and strand β1′ occupy in *Ab*ThiL. Strand β0 is unique to *Ab*ThiL and part of a loop in *Aa*ThiL. Starting with strand β2 secondary structure elements match up again.

All structures were refined with phenix.refine within Phenix^17^. Manual model building was done using Coot^18^. Ligand restraints were generated using Grade web server (http://grade.globalphasing.org) from GlobalPhasing Ltd. Molecular Replacement for the monoclinic crystal form was done with Phaser^19^ within the Phenix ligand pipeline. The quality of all structures was assessed using built-in tools in Coot, and using Molprobity^20^ via the Phenix interface. The dimer of AbThil is shown in Fig. 1.

Metals in the active site were modeled based on environment distances and geometry^21^, residual density, and anomalous difference density and validated using the CheckMyMetal server^22^. For anomalous difference density maps for data collected in-house at long wavelength, map intensities were compared with internal references, such as sulfur and phosphorous atoms from the substrates/produces using the following f″ for CuKa radiation: f″(Na) = 0.1e^−^, f″(Mg) = 0.2e^−^, f″(K) = 1.1e^−^, f″(Ca) = 1.3e^−^, f″(S) = 0.55e^−^, f″(P) = 0.43e^−^; for 0.97872 Å: f″(K) = 0.46e^−^, f″(Ca) = 0.57e^−^, and <0.1e^−^ for all others (http://skuld.bmsc.washington.edu/scatter/).

All metals with coordination distances of 2.1–2.2 Å were modeled as magnesium ions. Metal sites with coordination distances of 2.7–2.9 Å and strong 2Fo-Fc electron density were observed in the AMPPNP-bound (5CC8) and the AMPPNP/TMP-bound (5DD7) structures. A strong anomalous signal for both sites (13.4 and 8.3 sigma) in the AMPPNP-bound structure was additional evidence for potassium, even though no potassium was added during crystallization. By comparison the anomalous density for the phosphorous atoms in AMPPNP is slightly weaker with 7.9 sigma. The anomalous scattering coefficient at CuKα radiation is twice as high for potassium as it is for phosphorous. For the AMPPNP/TMP structure an anomalous signal could be detected for the potassium site, despite the weaker anomalous signal at shorter wavelength. For this structure, potassium was part of the crystallization condition.

**Figure 2:**
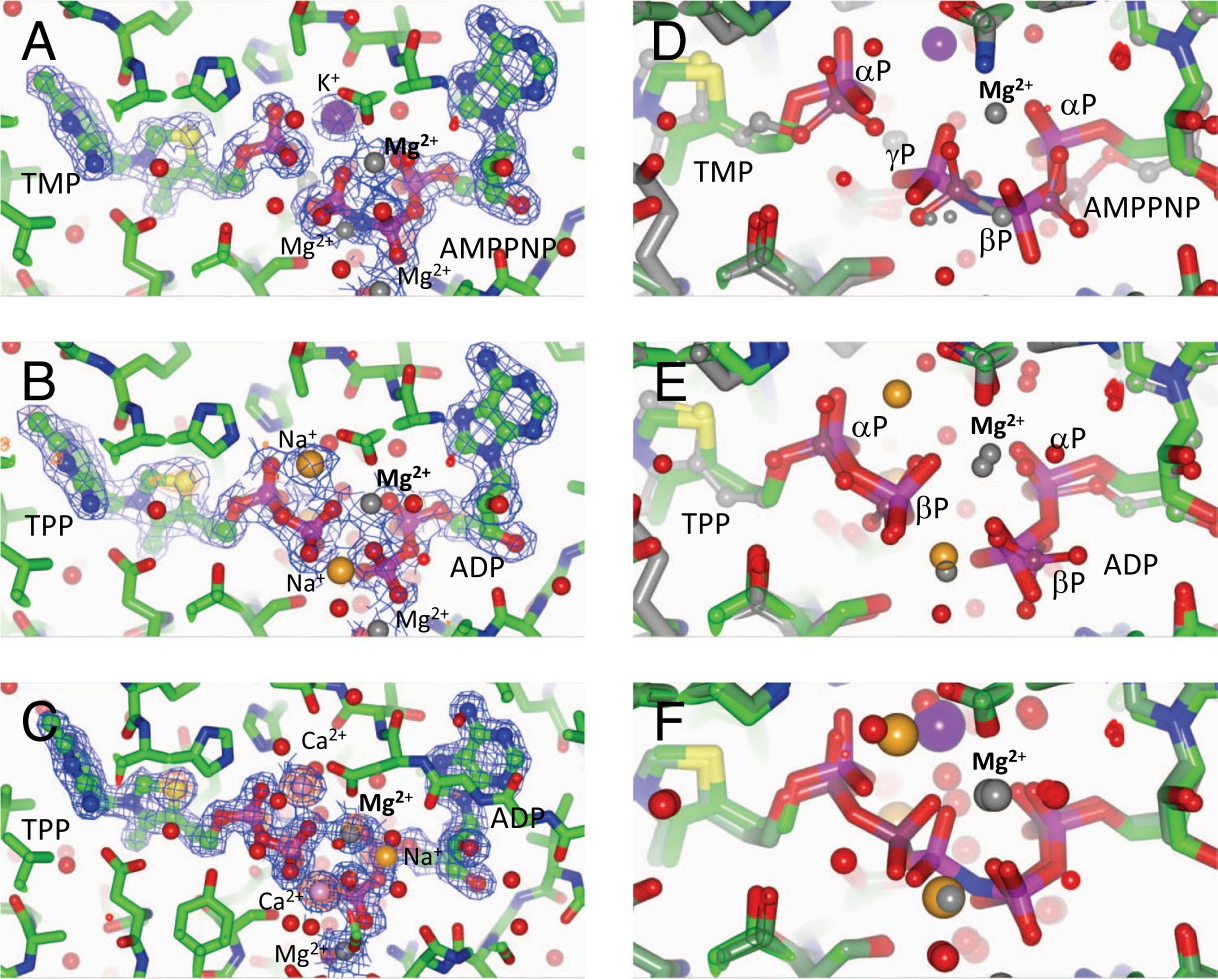
Active site of ThiL. Panels A–C show an overview of the active site and the bound ligands for *Ab*ThiL. Panels D–F zoom into the reaction site and compare *Ab*ThiL with *Aa*ThiL (**D**/**E**), and the product and substrate complex for *Ab*ThiL. (**F**) Metals are colored as follows: Mg^2+^ grey, Ca^2+^ pink, Na^+^ orange, K^+^ purple. (**A**) *Ab*ThiL with TMP and AMPPNP, oP form, 2Fo-Fc map at 1 σ (5DD7). (**B**) *Ab*ThiL with TPP and ADP, oP form, 2Fo-Fc map at 1 σ, anomalous map at 3 σ (5CM7). (**C**) *Ab*ThiL with TPP and ADP, mP form, 2Fo-Fc map at 1 σ, anomalous map^25^ at 3 σ (5D9U). (**D**) *Ab*ThiL with TMP and AMPPNP (ANP) in thick and bright, and *Aa*ThiL with TMP and AMPPCP (thin, dark, 3C9T), note different orientation of the α-phosphate groups of TMP. (**E**) *Ab*ThiL with ADP and TDP (thick, bright) and *Aa*ThiL with ADP and TPP (thin, dark, 3C9U), note the similar orientation of phosphate groups. (**F**) *Ab*ThiL with AMPPNP (ANP) and TMP (dark) and ADP and TPP (bright), suggesting an in-line transfer of the phosphate group. The invariable Mg^2+^ ion that either bridges the scissile phosphorester bond between β-phosphate and γ-phosphate in the substrate complex, or that bridges between β-phosphate of TPP and β-phosphate in ADP in the product complex is highlighted in bold.

For metal sites with coordination distances of ~2.4 Å, sodium and calcium were the most likely candidates. Sodium was modeled in the absence of an anomalous signal due to the low anomalous scattering coefficient (0.1e^−^). Since calcium has an anomalous signal at both wavelengths used, calcium was modeled if anomalous density could be detected, which was exclusively for the AMPNP/TMP data set (5DD7), even though no calcium was added to the crystallization set up. Metal ions in the immediate coordination sphere of substrates and products along with electron density are shown in Fig. 2A–C.

The coordinates and structure factors were deposited in the PDB with accession codes 5CC8, 5CM7, 5D9U, and 5DD7. Structure factors and anomalous differences for the iodide data set were deposited along with the corresponding AMPPNP-bound structure, 5CC8.

## Results and Discussion

### AbThiL crystallization and structure solution

*Ab*ThiL could be crystallized in a variety of conditions, all of which have in common a medium chain PEG as the precipitant and buffer in the pH range of 6.5–7.5. Two crystal lattices were observed, an orthorhombic crystal form, and a monoclinic crystal form. As part of the standard SSGCID pipeline, *Ab*ThiL was initially crystallized as apo protein and with AMPPNP/MgCl_2_. Diffraction was much better for nucleotide-bound protein. The structure was solved *de novo* using anomalous diffraction generated by soaking the crystals with iodide ions using a previously reported phasing protocol^23^.

### AbThiL structures

We obtained high-resolution structures, between 1.55 Å and 1.90 Å resolution, of *Ab*ThiL in complex with AMPPNP alone (orthorhombic), the substrate analogs AMPPNP/TMP (orthorhombic), and with the products ADP/TDP (orthorhombic and monoclinic) (Table 1). The orthorhombic crystal form was consistent for all complex structures and was refined for all ligands. The monoclinic data set for the ADP/TPP complex was refined in addition because of its significantly higher resolution.

All of the structures were well resolved: For the structures bound to the substrate/product pairs, residues Met1 or Ala2 through Phe304 or the C-terminal Ala305 could be modeled, while for the AMPPNP-bound structure residues Glu3 through Ile298 were modeled. All four models refined well with appropriate R-factors, no or few rotamer and Ramachandran outliers, and low Molprobity scores (Table 1).

In both crystal forms *Ab*ThiL crystallized as a dimer. The dimers of the various structures in this study were highly symmetric with RMSD between Cα atoms of the two chains within the dimer in the range of 0.2–0.3 Å for the orthorhombic structures, and 0.4 Å between the two chains of the monoclinic structure. Similarly, RMSD between protomers of different structures were small, 0.2–0.3 Å between chains A of the orthorhombic structures, and 0.4–0.5 Å between orthorhombic structures and the monoclinic structure. For consistency, the orthorhombic ADP/TDP-bound structure will be used as the reference.

### Comparison of AbThiL with folds of homologs

The closest structural homologs of *Ab*ThiLper PEBeFold/SSM^24^ search are ThiL proteins from *Methylobacillus flagellatus* (*Mf*ThiL, 44% identity, PDB ID 3MCQ; no primary citation) and from *Aquifex aeolicus* (*Aa*ThiL, 32% identity, PDB IDs 1VQV (apo, no primary citation, 3C9R, 3C9S, 3C9T, 3C9U^7^). RMSD for Cα atoms as determined by SSM are 1.3–1.4 Å for *Mf*ThiL and for various *Aa*ThiL structures.

The *Ab*ThiL structures assume the same two domain fold as *Mf*ThiL and *Aa*ThiLwith an N-terminal A domain and a C-terminal B domain. Since *Mf*ThiL was only crystallized as apo protein, this discussion will focus on the comparison between *Ab*ThiL and *Aa*ThiL^7^ and will follow the secondary structure nomenclature established for *Aa*ThiL, in which the short strand β0 is part of a loop.

In *Ab*ThiL, the A domain extends from the N-terminus to Gly138 and consists of the following sequence of secondary structure elements: α1, β0, β1, β2, α2, β3, α3, β4, β5, β6. Strands β2, β3, and β6 are each 11 residues long and form the core of twisted anti-parallel β-sheet, with further contributions from the shorter strands β4 and β5, and strands β0’ and β1′ from the other protomer. A loop comprising residues 137–149 bridges the two domains. The B-domain extends from Arg143 to the C-term and consists of the following sequence of secondary structure elements: β7, α4, α5, α6, β8, α7, β9, α8, α9, β10, α10, β11, β12, and β12. The core of this domain is a twisted mostly anti-parallel β-sheet that consists of strands β8, β10, β7, β11, β9, β12, and β13. In this sheet, strands β9 are β12 the only parallel strands Fig. 1A.

While there is a very good match of secondary structure elements between *Ab*ThiL and *Aa*ThiL, the N-terminal 30 residues are remarkably different (Fig. 1B,C). In *Aa*ThiL helix α1 (Phe9-Glu20) is adjacent to helix α3′ (Glu93-Tyr111) from the other protomer (Fig. 1C). In *Ab*ThiL, however, helix α1 (Glu3-Phe11) is adjacent to helix α3 (His93-Gly111) from the same protomer. The domain-swapped helix in *Aa*ThiL requires a sharp turn in a short loop (Tyr33-Lys36), just N-terminal of strand β2 (Lys37-Asn46). In *Ab*ThiL, this loop (Thr31-Asn34) is more extended and connects strands β1 (Ala28-Ile30) and β2 (Gln36-Val46) (Fig. 1B). In the structure of *Mf*ThiL, the N-terminus is oriented similar to the *Ab*ThiL structure. However, part of the protein chain of apo *Mf*ThiL occupies the ADP/ATP binding pocket. In contrast, the N-term of apo *Aa*ThiL superimposes with the N-term of ligand-bound *Aa*ThiL.

Interestingly, this N-terminal region of both *Aa*ThiL and *Ab*ThiL is involved in substrate binding: Ile23-Gly24-Asp25-Asp27 form a loop that interacts with the ribose-phosphate moiety of ADP/AMPPNP. Despite the domain swap this loop is conserved in sequence and structure between *Aa*ThiL and *Ab*ThiL. Helix α1 appears to be less conserved in structure and sequence between *Aa*ThiL and *Ab*ThiL despite a few hydrophobic interactions between residues from this helix and the adenine moiety of ADP/AMPPNP, see Fig. S2.

### Identification of metal-ion binding in the active site

In the apo structures of *Aa*ThiL (1VQV) and *Mf*ThiL (3MCQ), no metal ions had been modeled in the active site. In the ligand-bound *Aa*ThiL structures, metal ions were modeled in the active site, which suggests that substrate binding recruits metal ions. All metal ions were assigned as magnesium ions. However, while coordination distances for magnesium ions are expected to be close to 2.1 Å^21^, most of the coordination distances in the ligand-bound *Aa*ThiL structures are around 2.4 Å long which is unusually long for magnesium. The distances are more compatible with calcium, which was added as calcium chloride as a 10 mM additive to three of the structures (3C9R, 3C9S, 3C9T). In fact, for these three *Aa*ThiL structures, the analysis of the CheckMyMetal server^22^ suggests calcium or sodium as alternative metals. For the 3C9U, which was crystallized without additional calcium chloride, some metals are likely magnesium ions, while others are likely to be calcium or sodium ions. At the time of the publication of the *Aa*ThiL structures, sophisticated and convenient metal validation tools, such as the CheckMyMetal server, were not available yet.

For the *Ab*ThiL structures, both high resolution data, anomalous diffraction, and the availability of metal validation tools allows us to accurately identify metals in the active site. Our analysis shows that the active site can accommodate a variety of metals, and that the metal content is in part influenced by the crystallization condition, see Fig. 2 and Table 2. Metal ions that bridge two phosphate groups of AMPPNP or ADP, that bridge the AMPPNP/TMP or ADP/TPP, respectively, or that bridge the two phosphate groups of TPP are summarized in Table 2. Despite the variability of metal content, it appears as if in this set of structures adenosine phosphates have a strong preference for magnesium. One metal that is in the center of the reaction is consistently magnesium: In the substrate complex a magnesium ion complexes both the scissile phosphoester bond in ATP, γ-phosphate and β-phosphate, and also the uncleaved β-phosphate and α-phosphate. The equivalent metal in the product complex is a magnesium ion as well, bridging between β-phosphate of TPP and β-phosphate in ADP and between α-phosphate and β-phosphate of ADP. In contrast, the phosphoester bond that is formed in the reaction, between α-phosphate and β-phosphate of TPP can be complexed either by magnesium or calcium.

**Table 2:** Metal coordination of adenosine phosphate and thymidine phosphate groups in various structures of *Ab*ThiL and *Aa*ThiL.

Data set	*Ab*ThiL AMPPNP, oP	*Ab*ThiL AMPPNP-TMP, oP	*Ab*ThiL ADP/TDPTPP, oP	*Ab*ThiL ADP-/TPDP, mP	*Aa*Thil AMPPCP/TMP	*Aa*ThiL ADP/TPP,
PDB code	5CC8	5DD7	5D9U	5CM7	3C9T	3C9U
Adenosine Pα-Pβ	Mg^2+^	Mg^2+^	Mg^2+^	Mg^2+^		
Adenosine Pβ-Pγ	Mg^2^/Mg^2+^	Mg^2+^/Mg^2+^	n/a ^+^	n/a	–/Mg^2+^	n/a
Adenosine/Thymidine bridge	n/a	Mg^2+^/Mg^2+^	Mg^2^/Mg^2+^	Mg^2^/Ca^2+^	Mg^2+^	Mg^2+^/Mg^2+^
Thymidine Pα-Pβ	n/a	n/a	Mg^2^/Mg^2+^	Ca^2^/Ca^2+^	n/a	Mg^2 +^

While there is some plasticity in the metal content, several metal positions appear to be conserved between the structures presented here.

### Ligand binding site

Ligand binding has been comprehensively described for *Aa*ThiL^7^. In brief: The active site is located in the dimer interface. ATP/ADP is deeply buried in pocket generated at the interface between molecule A and B, including the N-terminal helix. The adenine moiety is bound in a hydrophobic pocket that is rich in conserved isoleucine and valine residues. The TMP/TPP binding site is exclusively formed by one subunit, however at the interface between the two domains. The pyrimidine ring of TMP/TDP is exposed to the solvent.

The location of the substrates is highly conserved between *Aa*ThiL and *Ab*ThiL, Fig. 2. Due to the different arrangement of helix α1 in *Ab*ThiL, ATP/ADP bind to one molecule of the dimer, while TMP/TDP bind to the other molecule of the dimer. The binding pocket for TMP/TDP is very conserved between *Aa*ThiL and *Ab*ThiL. Due to the reorientation of helix α1, the binding pocket for ATP/ADP has some significant changes for residues in helix α1, while maintaining a very similar hydrophobic pocket.

While most of the phosphate groups of equivalent complexes in *Aa*ThiL and *Ab*ThiL are structurally similar, the TMP phosphate group deviates significantly, Fig. 2D,E. An explanation for this is not obvious.

The substrate- and product-bound structures of *Ab*ThiL allow us an insight in a probable reaction mechanism. An in-line attack of the α-phosphate group of TMP on the γ-phosphate group of ATP had been postulated for *Aa*ThiL^7^. Similarly, in the *Ab*ThiL substrate complex (AMPPNP/TMP, Fig. 2D), the α-phosphate group of TMP is already positioned for an in-line attack on the non-hydrolyzable γ-phosphate group of AMPPNP. In addition, we observed that the product complex (ADP/TPP, Fig. 2E) has a very similar conformation as the substrate complex. A superposition of the product complex and the substrate complex of *Ab*ThiL (Fig. 2F) highlighs that the α-phosphate of TMP/TPP, and the α-phosphate and the β-phosphate groups of AMPPNP/ADP virtually do not move. Even the transferred phosphate group, the γ-phosphate in AMPPNP and the β-phosphate in TPP, barely changes position. The distance between the phosphorous atoms is only 1.0 Å.

In summary, this set of high resolution *Ab*ThiL structures in complex with substrate analogs and products provides a very detailed view of the active site. We could identify a variety of metals complexing the products and substrates, while the transferred phosphate groups appear to be specific for magnesium ions. Furthermore, the structures provide further evidence for the proposed in-line attack reaction mechanism.

## Supplementary information

Supplementary figures 1 and 2

## References

[1] Settembre E, Begley TP, Ealick SE (2003). Structural biology of enzymes of the thiamin biosynthesis pathway. Current opinion in structural biology.

[2] Webb E, Downs D (1997). Characterization of thiL, encoding thiamin-monophosphate kinase, in Salmonella typhimurium. The Journal of biological chemistry.

[3] Edwards TE, Ferré-D’Amaré AR (2006). Crystal structures of the thi-box riboswitch bound to thiamine pyrophosphate analogs reveal adaptive RNA-small molecule recognition. Structure (London, England: 1993).

[4] Rice LB (2008). Federal funding for the study of antimicrobial resistance in nosocomial pathogens: no ESKAPE. The Journal of infectious diseases.

[5] Peleg AY, Seifert H, Paterson DL (2008). Acinetobacter baumannii: emergence of a successful pathogen. Clinical microbiology reviews.

[6] Gallagher LA (2015). Resources for Genetic and Genomic Analysis of Emerging Pathogen Acinetobacter baumannii. Journal of bacteriology.

[7] McCulloch KM, Kinsland C, Begley TP, Ealick SE (2008). Structural studies of thiamin monophosphate kinase in complex with substrates and products. Biochemistry.

[8] Aslanidis C, de Jong PJ (1990). Ligation-independent cloning of PCR products (LIC-PCR). Nucleic acids research.

[9] Choi R (2011). Immobilized metal-affinity chromatography protein-recovery screening is predictive of crystallographic structure success. Acta crystallographica. Section F, Structural biology and crystallization communications.

[10] Bryan CM (2011). High-throughput protein production and purification at the Seattle Structural Genomics Center for Infectious Disease. Acta crystallographica. Section F, Structural biology and crystallization communications.

[11] Kabsch W (2010). XDS. Acta crystallographica. Section D, Biological crystallography.

[12] Grosse-Kunstleve RW, Adams PD (2003). Substructure search procedures for macromolecular structures. Acta crystallographica. Section D, Biological crystallography.

[13] Read RJ, McCoy AJ (2011). Using SAD data in Phaser. Acta crystallographica. Section D, Biological crystallography.

[14] The CCP4 suite: programs for protein crystallography. *Acta crystallographica. Section D, Biological crystallography* **50**, 760–3 (1994). 10.1107/S0907444994003112 15299374

[15] Cowtan K (2010). Recent developments in classical density modification. Acta crystallographica. Section D, Biological crystallography.

[16] Langer G, Cohen SX, Lamzin VS, Perrakis A (2008). Automated macromolecular model building for X-ray crystallography using ARP/wARP version 7. Nature protocols.

[17] Adams PD (2010). PHENIX: a comprehensive Python-based system for macromolecular structure solution. Acta crystallographica. Section D, Biological crystallography.

[18] Emsley P, Lohkamp B, Scott WG, Cowtan K (2010). Features and development of Coot. Acta crystallographica. Section D, Biological crystallography.

[19] McCoy AJ (2007). Phaser crystallographic software. Journal of applied crystallography.

[20] Chen VB (2010). MolProbity: all-atom structure validation for macromolecular crystallography. Acta crystallographica. Section D, Biological crystallography.

[21] Zheng H, Chruszcz M, Lasota P, Lebioda L, Minor W (2008). Data mining of metal ion environments present in protein structures. Journal of inorganic biochemistry.

[22] Zheng, H. *et al*. CheckMyMetal: A macromolecular metal-binding validation tool. *Acta Crystallographica Section D: Structural Biology*10.1107/S2059798317001061 (2017). 10.1107/S2059798317001061 PMC5349434 28291757

[23] Abendroth J (2011). SAD phasing using iodide ions in a high-throughput structural genomics environment. Journal of structural and functional genomics.

[24] Krissinel E, Henrick K (2004). Secondary-structure matching (SSM), a new tool for fast protein structure alignment in three dimensions. Acta crystallographica. Section D, Biological crystallography.

[25] Gouet P, Robert X, Courcelle E (2003). ESPript/ENDscript: Extracting and rendering sequence and 3D information from atomic structures of proteins. Nucleic acids research.

