# Supplementary material for: Crystal structures of thiamine monophosphate kinase from Acinetobacter baumannii in complex with substrates and products
Source: Sci Rep. 2019 Mar 13;9:4392. doi: 10.1038/s41598-019-40558-x (PMC6416309; doi:10.1038/s41598-019-40558-x)
Supplement: Supplementary file 1 — Supplementary figures 1 and 2 [file 41598_2019_40558_MOESM1_ESM.pdf]

Crystal structures of thiamine monophosphate kinase from *Acinetobacter baumannii* in complex with substrates and products

Amy H. Sullivan[1,2], David M. Dranow[1,2], Peter S. Horanyi[1,2], Donald D. Lorimer[1,2], Thomas E. Edwards[1,2], Jan Abendroth[1,2, *]

Affiliations

1) UCB/Beryllium Discovery, 7869 NE Day Road West, Bainbridge Island, WA, USA

2) Seattle Structural Genomics Center for Infectious Disease, Seattle WA, USA

* corresponding author: jan.abendroth@ucb.com

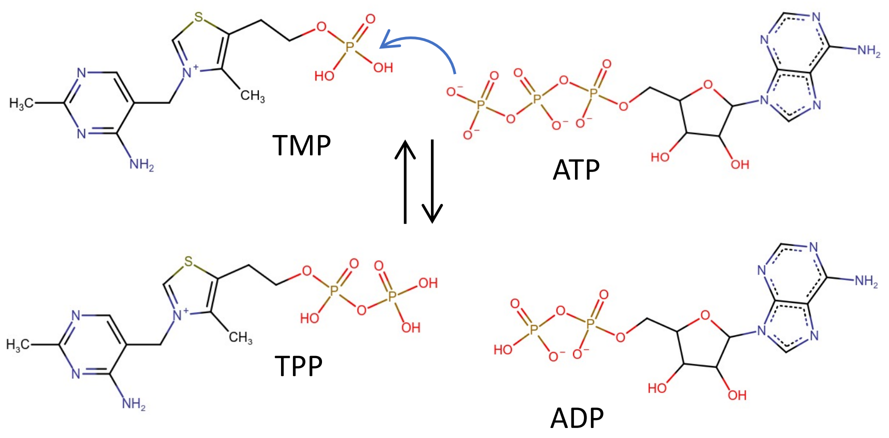

Figure S1:
A schematic representation of the reaction carried out by ThiL, the conversion of TMP and ATP to TPP and ADP.

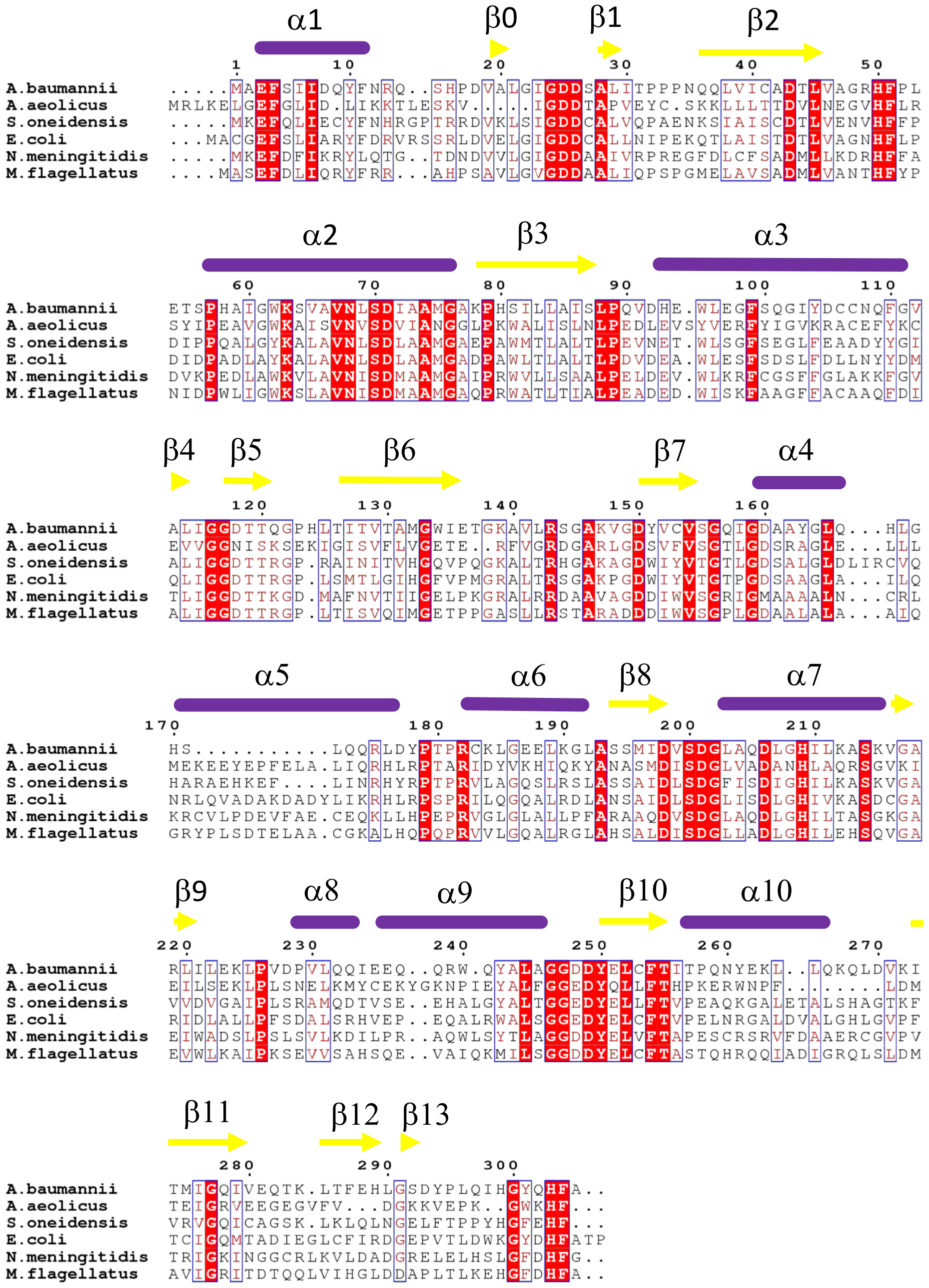

Figure S2:

Multiple sequence alignment of *Ab*ThiL with *Aa*ThiL, along with *Shevanella oneidensis*, *Eschericia coli*, *Neisseria meningitidis*, *Methylobacillus flagellatus*. The Figure was prepared with ESPript [25]
